# The Impact of the Clinical Pharmacist-Led Diabetes Education on the Knowledge and Attitude of Individuals with Type II Diabetes Mellitus: An Interventional Study

**DOI:** 10.3390/pharmacy12050151

**Published:** 2024-09-29

**Authors:** Safaa Badi, Sara Zainelabdein Suliman, Rayan Almahdi, Mohammed A. Aldomah, Habab Khalid Elkheir, Mohamed Izham Mohamed Ibrahim, Mohamed H. Ahmed

**Affiliations:** 1 Department of Clinical Pharmacy, Faculty of Pharmacy, Omdurman Islamic University, Khartoum 14415, Sudan; sarazainabideen@gmail.com (S.Z.S.); rayan94.malik@gmail.com (R.A.); mohammedalshuraney@gmail.com (M.A.A.); hababelkheir@smcreg.gov.sd (H.K.E.); 2 Department of Clinical Pharmacy, University of Science and Technology, Khartoum 14411, Sudan; 3 Department of Clinical Pharmacy and Practice, College of Pharmacy, QU Health, Qatar University, Doha P.O. Box 2713, Qatar; mohamedizham@qu.edu.qa (M.I.M.I.); 4 Department of Medicine and HIV Metabolic Clinic, Milton Keynes University Hospital NHS Foundation Trust, Eagle Stone, Milton Keynes, Buckinghamshire MK6 5LD, UK; mohamed.hassan-ahmed@mkuh.nhs.uk; 5 Department of Geriatric Medicine, Milton Keynes University Hospital NHS Foundation Trust, Eagle Stone, Milton Keynes, Buckinghamshire MK6 5LD, UK; 6 Honorary Senior Lecturer of the Faculty of Medicine and Health Sciences, University of Buckingham, Buckinghham MK18 1EG, UK

**Keywords:** clinical pharmacist, education, knowledge, attitude, diabetes mellitus

## Abstract

Background: Diabetes mellitus is a complex condition to manage. Patients with a greater understanding and knowledge of their condition might achieve better glycemic control than others. This study aimed to evaluate the impact of clinical pharmacist-led diabetes education on the knowledge and attitude of individuals with type II diabetes mellitus (T2DM). Methods: This study was a quasi-experimental study which was conducted at a diabetes clinic in Khartoum, Sudan. The study population was adult individuals with T2DM who attended the diabetes clinic. The estimated sample size was 182 participants. The participants were selected randomly by a simple random sampling method. The knowledge and attitudes of the participants were assessed at baseline and at the end of the study after 12 months. The intervention was carried out through educational materials about diabetes and medications for its treatment. Results: The majority of the participants were females. The mean age was 54.5 (±10) years. Most participants had a family history of diabetes (69.2%). The mean knowledge score after the intervention was increased by 1.4 (±0.1) from baseline, *p* value (<0.001), while the mean attitude score was increased by 1.7 (±0.2) from baseline, *p* value (<0.001). At baseline, 14.8% of the participants had a high level of knowledge and 18.7% had a negative attitude, while after intervention for 12 months, 28.5% of them had a high level of knowledge and 16.8% had a negative attitude (*p* values < 0.001, 0.032, respectively). Conclusions: The knowledge of and attitudes towards diabetes differed significantly as a result of the educational program provided by the clinical pharmacist.

## 1. Introduction

Due to the increase in the number of individuals with type 2 diabetes (T2DM) worldwide, there may be a rising economic burden from the disease globally [1,2]. It is concerning that despite clinical and financial efforts by organizations and individuals worldwide, the incidence and prevalence of T2DM continue to rise. Additionally, there has been an increase in premature deaths due to T2DM [2]. The World Health Organization (WHO) has identified several factors that increase susceptibility to developing T2DM, such as a family history of diabetes, a sedentary lifestyle, obesity, age, a history of gestational diabetes, cardiovascular disease and its risk factors, and certain ethnicities (South Asian, Afro-Caribbean, Hispanic). Importantly, T2DM is characterized by a slow deterioration towards hyperglycemia. A considerable number of patients present with complications at diagnosis, resulting from a long-standing uncontrolled hyperglycemic state [3]. T2DM is a complex condition to manage as it is related to several important factors such as food culture, physical activity, the psychological status and motivation of individuals, and the availability and affordability of health care [4]. Educating patients with T2DM about their disease is crucial. Individuals with diabetes who are knowledgeable about the disease may achieve better glycemic control than others. Unfortunately, proper health education to prevent and treat diabetes in Sudan and developing countries is lacking, and significant gaps remain. For instance, the Chennai Urban Rural Epidemiology Study (CURES) in India showed that, among individuals with diabetes, 41.0% were aware that diabetes could be prevented, and 40.6% were aware that diabetes could cause complications. The same study revealed that, among the entire population recruited, only 11.9% were aware that obesity and physical inactivity are risk factors for diabetes. The authors concluded that there is a need for health education about diabetes in both rural and urban areas of India [5]. Furthermore, a cross-sectional study conducted with 906 Saudi individuals with diabetes, recruited from 47 hospitals across 8 cities, revealed a significant lack of knowledge regarding diabetes mellitus. However, the participants were well-informed about its implications and the necessary precautions [6]. In the United Arab Emirates, among 575 individuals with diabetes, 31% had poor knowledge of diabetes, 72% had negative attitudes towards having the disease, and 57% had HbA1c levels reflecting poor glycemic control. Only 17% had adequate blood sugar control, while 10% admitted to non-compliance with their medications. Despite the knowledge, practice, and attitude scores being statistically significantly positive, none of these scores were significantly correlated with HbA1c levels [7]. However, studies in India revealed that while the general population’s knowledge about diabetes was good, their practices were substandard [8,9]. This may suggest the need for further research into the community’s inclination toward using herbal medicine rather than antidiabetic medication, as well as incorrect beliefs about certain medications (e.g., the belief that statins may cause paralysis). These factors also need to be researched in Sudan in the future. Diabetes education in Sudan can play an important role in reducing the risk of developing diabetes complications. This is not only the responsibility of physicians or diabetes specialists but also includes other health care professionals, such as clinical pharmacists. Proper patient health education about the significance of lifestyle changes and diabetes care may help achieve good glycemic control. Saudi Arabia is known for the progressive rise in the prevalence of T2DM, and health authorities have recommended the need to organize health education programs for the general population to change poor practices and attitudes toward mitigating the risk associated with the development of diabetes [1,2,3]. However, other studies have indicated that people with T2DM possess good knowledge, a positive attitude, and effective practices. For instance, a cross-sectional study recruited 18,697 adults (aged 18 years and above; 7796 males and 10,901 females; 6780 non-diabetic and 11,917 with T2DM) in Bangladesh. The average knowledge score among T2DM subjects was 68%, and the attitude score was 67%. The knowledge, attitude, and practice scores were found to be significantly higher in those with diabetes compared to those without diabetes. Males with diabetes showed significantly higher scores in knowledge and practice regarding diabetes, while females with diabetes showed significantly better attitudes towards the condition. Importantly, knowledge scores were strongly correlated with education, income, residence, diabetic status, body mass index, and attitude [9]. Among 149 individuals with T2DM in South Africa, 64.4% were obese and 24.0% were overweight, despite having good knowledge of lifestyle modifications. While 63.4% claimed to exercise regularly, two-thirds monitored their weight irregularly [10]. Niroomand et al. showed that a diabetes education program improved knowledge, attitude, and practice among 200 individuals with diabetes in Iran. Their data indicated good knowledge in 61.41% of participants, a positive attitude in 50.44%, and good practice in 52.23% [11]. A cross-sectional study conducted in Saudia Arabia revealed a favorable level of knowledge, behavior, and practices among participants with type 2 diabetes [12]. Importantly, Al-Wagdi and Al-Hanawi concluded that high knowledge scores about diabetes will not improve attitudes and practices related to diabetes [13]. Empowering patients with diabetes awareness may help reduce microvascular problems, such as visual impairment and blindness caused by diabetic retinopathy.

For example, studies have indicated that patients’ knowledge regarding diabetic retinopathy was poor, and a lack of awareness of the necessity of retinal screening was the greatest barrier to regular screening [14,15]. However, other studies showed good average knowledge and practice regarding diabetic retinopathy and diabetes-related complications [16,17,18]. Individuals’ nutritional knowledge and attitudes play an important role in diabetes management. As a result, governmental and non-governmental organizations must provide health education on food management policies for T2DM patients and make dietitian services available to all people with diabetes. Dietitians can also assist in preventing diabetes-related problems, preparing diet plans, and offering education on the necessity of a diabetes diet [19,20]. It has been shown that there is a gap between patients’ dietary knowledge/attitude and practices [21]. This is an important challenge for clinical pharmacists and dietitians in Sudan, as most of the traditional and local foods and their impact on diabetes remain to be researched. The focus of clinical pharmacists in Sudan should be to improve knowledge about diabetes and ultimately improve attitudes and practices using simple and advanced means of health education. Moreover, intensive education on diabetes medications from both community and clinical pharmacists is crucial to improve the knowledge of individuals about diabetes and enhance their attitudes [22]. Some studies have found that patients showed inadequate knowledge regarding diabetes and the self-administration of insulin and other medications [23,24]. On the other hand, it is possible to achieve good knowledge about and attitudes towards diabetes medication adherence [25,26]. However, many Sudanese patients are non-adherent to their medications [27] and have insufficient knowledge regarding diabetes mellitus [28]. In addition, the high cost associated with diabetes services, especially in developing countries, means it will be cheaper and more practical to invest in health education. In the presence of an extreme shortage of diabetes specialists in Sudan, trained clinical pharmacists may be able to help improve health education about diabetes. Therefore, the aim of the current study is to explore the potential impact of pharmacist-led educational interventions on the knowledge and attitudes of the participants regarding diabetes mellitus.

## 2. Methodology

### 2.1. Study Setting

This study was a quasi-experimental study, which took took place at a diabetes clinic at Omdurman Military Hospital (OMH), and recruited individuals with T2DM. The data were collected over 12 months from 2021 until 2022.

### 2.2. Selection Criteria

Individuals with T2DM aged more than 18 years (adult, male or female, older adult) were eligible for the study. Pregnant women with diabetes, patients taking immunosuppressants or antidepressants that may affect blood glucose levels at the time of the study, and any patients who were unable to communicate were excluded from the study.

### 2.3. Sample Size and Sampling Techniques

The sample size was calculated using an online sample size calculator, and individuals were selected randomly by a simple random sampling method [29]. The estimated sample size was 161 individuals with diabetes mellitus but we recruited 182 participants to account for dropouts.

### 2.4. Data Collection Tools and Method

Pre-structured, standardized questionnaires were used to collect the participants’ data [30,31]. The questionnaires consisted of four sections; section one included the socio-demographic data (including phone numbers, gender, age, marital status, residence, and educational level) and section two included the medical history of the participants. The third section consisted of questions assessing the participants’ knowledge about diabetes, lifestyle changes, diabetes complications, and diabetes medications and the fourth section included questions to assess the attitudes of the participants towards diabetes mellitus.

### 2.5. Methods of Calculating Scores of Knowledge and Attitude

#### 2.5.1. Knowledge Score

The knowledge assessment component had 20 questions with yes, no, and I don’t know responses; each correct answer was given one point, while each incorrect answer or I don’t know response was given zero. Knowledge scores ranged from 0 to 20. The median knowledge scores were categorized into three categories based on their quartiles. Those who scored less than quartile 1 (Q1; 25th) were classified as having a low degree of knowledge. Those who scored above quartile 3 (75th) were classified as having a high level of knowledge. Others were classified as having a moderate degree of knowledge if their score was between Q1 (25th) and Q3 (75th).

#### 2.5.2. Attitude Score

The attitude questions comprised 14 questions with three responses: agree, neutral, and disagree. A response that indicated a positive attitude scored two points, whereas a response that indicated a negative attitude scored zero points. A neutral response was given one point. The total attitude scores ranged between 0 and 28, with higher scores indicating a positive attitude. The median attitude scores have been divided into three categories based on their quartiles; those who scored less than Q1 (25th) were defined as having a negative attitude, while those who scored higher than Q3 (75th) were classified as having a positive attitude. The others were regarded as having a fair attitude if their score was between Q1 (25th) and Q3 (75th).

### 2.6. Phases of the Data Collection

#### 2.6.1. Phase One (Selection of the Participants)

This phase was allocated to selecting the participants, and it took three months.

#### 2.6.2. Phase Two: (Baseline Assessment)

This phase was allocated to filling out the questionnaires; baseline data were recorded, and detailed information was taken from the participants.

#### 2.6.3. Phase Three: Intervention (Education)

In this phase, educational materials about diabetes mellitus were provided to the participants in the form of twelve videos sent within a period of five months.

In those videos, we provided detailed information about diabetes such as diabetes mellitus pathophysiology, blood glucose goals, and glycosylated hemoglobin (HbA1c) goals, and information about the management of hyperglycemic and hypoglycemic episodes and how to avoid them. As well as information regarding diabetes mellitus medications.

A total of twelve videos (with average 3–5 min duration) were provided to the participants during their monthly visits to the clinic. The videos were scheduled more frequently during the early months of the interventional period (5 months) to ensure patient engagement and provide enough opportunities and time to address all patients’ goals and concerns. Three videos were sent to the participants in the first and second months, and two videos in each of the remaining three months. The average length of the visits was about 15–20 min. The videos were distributed to all participants.

### 2.7. Ethical Approval

The ethical approval was obtained from Omdurman Islamic University (OIU/FPGS-January 2020) and Omdurman Military Hospital (GDMS/April 2021) and the study was conducted in line with the principles of the Declaration of Helsinki.

### 2.8. Data Analysis

Data were analyzed by using the Statistical Package for the Social Sciences (SPSS) version 28. Descriptive statistics were used to describe the data and inferential statistics were performed to determine any significant differences between the outcome variables at two points. The paired sample *t*- test and McNemar test were carried out to compare the outcomes before and after the intervention. A *p*-value less than 0.05 was considered significant.

## 3. Results

### 3.1. Socio-Demographic Characteristics of the Participants at Baseline

The sample size in this study was 182 participants. A total of 80.8% (*n* = 147) of the participants were females. The mean age was 54.5 (±10) years. Most participants had a family history of diabetes (69.2%). Nearly all participants in this study have never been alcoholic abusers (96.7%) or smokers (93.4%). More than 50% of participants suffered from episodes of recurrent hyper or/and hypoglycemia, retinopathy, and peripheral neuropathy. Tooth decay was the most reported complication, which affected (70.9%) of the participants (Table 1).

### 3.2. Knowledge of the Participants towards Diabetes Mellitus at Baseline

More than 60% of the participants in this study were knowledgeable about the symptoms and complications of hyperglycemia. In comparison, most of them (more than 90%) were knowledgeable about the role of lifestyle modifications in lowering blood glucose levels (Table 2).

### 3.3. Participants’ Attitude towards Diabetes at Baseline

Of the patients, 71.4% (n = 130) % were not afraid of diabetes. A total of 82.1% (n = 150) of them felt satisfied with their lives. More than two-thirds of them reported that they could control their blood glucose and weight and make lifestyle changes. More than two-thirds of the participants reported that they could control their blood glucose and weight and make lifestyle changes (Table 3).

### 3.4. Knowledge, Attitude Scores, and HbA1c Levels at Baseline and at 12 Months

The paired sample *t*-tests revealed that the average scores for knowledge and attitude were comparable between the two time periods; we found that the mean knowledge score at baseline was 14.8 (±2.5), while it was 16.2 (±1.9) after intervention with the mean difference of 1.4 (±0.1), *p* value (<0.001). Furthermore, the average scores for attitude was 21.7 (±3.3) at baseline, while it was 23.4 (±2.9) after the intervention with a mean difference of 1.7 (±0.2), *p* value (<0.001). The McNemar test results revealed that, at baseline, 14.8% of the participants had a high level of knowledge and 18.7% had a negative attitude, while after intervention for 12 months, 28.5% of them had a high level of knowledge and 16.8% had a negative attitude (*p* values < 0.001, 0.032, respectively). The mean HbA1c level decreased from 8.7 (±2.2)% at baseline to 6.8 (±0.8)% at the end of the study (*p* value < 0.001) (Table 4).

## 4. Discussion

Patient education can include providing both pharmacological and non-pharmacological information to patients and their caregivers. A clinical pharmacist is considered an essential member of multidisciplinary teams [32]. Individuals with diabetes need direct and comprehensive information about managing their condition, both pharmacologically and non-pharmacologically. Significant gaps exist in knowledge about T2DM, as individuals with diabetes generally have a poor understanding of their condition, especially in the rural areas of Sudan [28].

In our study, at baseline, 14.8% of the subjects had a high level of knowledge. Furthermore, Adam et al. showed, in Sudanese individuals with T2DM, that majority of the patients demonstrated good dietary knowledge (54.6%), a positive attitude (79%), and good practice (58%). They showed education regarding diet will lead to a significant increase in knowledge. The combination of good knowledge and a positive attitude result in an improvement in dietary habits [33]. Similar observations were made by Al-Maskari et al. in the United Arab Emirates (UAE), as they reported a significant positive increase in knowledge, practices, and attituded towards diabetes [7]. Farag et al. showed that, among Sudanese individuals with diabetes, 51.4% had sufficient knowledge about diabetes and its complications, while 48.6% had insufficient knowledge. Furthermore, their data showed that these patients had sufficient knowledge about foot examinations, inspections of shoes, and diet. However, knowledge about exercise and eye examination was insufficient [30]. In Fiji, Zibran and Mohammadnezhad found that the mean knowledge (23.3/30), attitude (23.1/30), and practice (7.1/10) levels were high among individuals with T2DM. A significant association with high levels knowledge was found for those individuals with a university degree, who were employed, and who had a high average monthly income [34]. Furthermore, our results differ from those of Farag and Zibran and Mohammadnezhad [30] which found that more than 50% of participants had good knowledge. These discrepancies may be due to differences in the methodology or the knowledge scoring methods used. Importantly, we noticed an improvement in the level of knowledge at the end of our study to 28.5%. This endorses the valuable role of clinic pharmacists in diabetes care. The involvement of clinical pharmacist in diabetes care was shown to increase patients’ knowledge and participation in decision-making regarding the therapeutic plan [35].

Additionally, well-designed educational programs tailored to the needs of the community, including individuals living with diabetes, could significantly improve knowledge and change attitudes. Other studies have shown poor knowledge and unsatisfactory practices and attitudes among T2DM patients [3,4,5,6,8]. The overall mean knowledge score for the participants in our study improved by 1.4 points from baseline to the study’s end (*p* < 0.001). Similar observations were reported in other studies. For example, George et al. from India reported that the knowledge score increased by the end of the study [36]. Rothman et al., from the United States, also reported that the intervention group showed more significant improvements in diabetes knowledge [37]. Malathy et al., from India, found that the overall knowledge, attitude, and practice (KAP) scores of the interventional group were significantly higher [38]. A Jordanian study by Wishah et al. reported a significant improvement in the mean knowledge score of the intervention group compared to the control group [39]. These results indicate that knowledge among the interventional group increased significantly from baseline.

On the other hand, intensive education regarding diabetes medications by both community and clinical pharmacists may directly lead to improvements in patients’ attitudes and practices [40]. Our study found that the attitudes of participants improved positively as the study progressed, compared to their baseline attitudes. This result aligns with the findings of Malathy et al. [38], who reported that the mean attitude score in the test group increased by one point from baseline to the study’s end (*p* < 0.001). Moreover, our study found that slightly more than one-fifth of the participants had a positive attitude at baseline. Importantly, positive attitude towards diabetes were observed in 28% of subjects in the UAE [7]. At the study’s end, the mean attitude score among the participants increased by 1.7 points from baseline. These results indicate that the attitudes among the participants significantly improved from baseline to the study’s end. The study may pave the way for the involvement of clinical pharmacists in diabetes management, particularly in low-resource settings, through diabetes counseling [41]. The need for health education for individuals with diabetes was emphasized in different studies [9,11,33]. This could help increase knowledge, practices, and attitudes toward diabetes and may also reduce the risk of developing diabetes complications in the future. Clinical pharmacists can be involved in diabetes management both in outpatient and inpatient settings. Their role will be most effective if they work in close collaboration with diabetes specialists and physicians [42,43,44,45].

The study is not without limitations, as participants were recruited solely from the capital, Khartoum. In addition, 80.8% of the participants were female. Therefore, future studies involving different centers across Sudan with the recruitment of equal numbers of males and females will allow for the findings to be generalized and applied to the entire country. Furthermore, in this study we did not consider a control group as the involvement of a control group may have reflected a more prominent effect of the clinical pharmacist’s intervention.

Future research may also assess the role of diabetes dietitian specialists and a multidisciplinary approach in improving patients’ knowledge, attitudes, and practices.

## 5. Conclusions

The knowledge and attitude towards diabetes improved significantly as result of an educational program provided by a clinical pharmacist.

## Figures and Tables

**Table 1:** Baseline and clinical information of the participants (n = 182).

Socio-Demographic Characteristics	Responses	N	%
Gender	Males	35	19.2
	Females	147	80.8
Age/Years	Mean (±SD)	54.5 (±10)	
Marital status	Married	149	81.9
	Unmarried	5	2.7
	Divorced	7	3.8
	Widowed	21	11.5
Residence	Urban	165	90.7
	Rural	17	9.3
Educational level	Illiterate	26	14.3
	Primary school	51	28.0
	Secondary school	73	40.1
	Bachelor degree	29	15.9
	Postgraduate level	3	1.6
BMI	Mean (±SD)	29.4 (±6.3)	
FH of diabetes	-	126	69.2
Alcohol use	Never	176	96.7
	I stopped	6	3.3
Smoking	Never	170	93.4
	Sometimes	1	0.5
	I stopped	10	5.5
	Yes (1 cig/day)	1	0.5
Complications	History of renal diseases	11	6.0
	History of cardiac diseases	19	10.4
	Episodes of recurrent hyper or/and hypoglycemia	105	57.7
	Tooth decay	129	70.9
	Peripheral neuropathy	94	51.6
	Retinopathy	104	57.1
	History of renal diseases	11	6.0

SD: standard deviation; BMI: body mass index; FH: family history. Cardiac diseases Ischemic heart diseases, heart failure, heart valves replacement, stroke, TIA, atherosclerosis. Renal diseases: renal stones, acute kidney injury, chronic kidney diseases, benign prostatic hyperplasia.

**Table 2:** Diabetes-related knowledge questions at baseline (n = 182).

	Yes	No	DN
1. Diabetes mellitus is a hereditary disease	101 (55.5)	53 (29.1)	28 (15.4)
2. Diabetes mellitus is a curable disease	52 (28.6)	120 (65.9)	10 (5.5)
3. Increased thirst is a sign of hyperglycemia	143 (78.6)	34 (18.7)	5 (2.7)
4. Frequent skin and urinary infections are signs of hyperglycemia	134 (73.6)	37 (20.3)	11 (6)
5. Ophthalmic problems are a complication of hyperglycemia	154 (84.6)	21 (11.5)	7 (3.8)
6. Foot infections are a complication of hyperglycemia	147 (80.8)	30 (16.5)	5 (2.7)
7. Cardiac problems are a complication of hyperglycemia	84 (46.2)	52 (28.6)	46 (25.3)
8. Renal problems are a complication of hyperglycemia	110 (60.4)	33 (18.1)	39 (21.4)
9. Neuropathy is a complication of hyperglycemia	152 (83.5)	24 (13.3)	6 (3.3)
10. Can the drugs be stopped, if diabetes is controlled?	82 (45.1)	97 (53.3)	3 (1.6)
11. Dietary modification can control diabetes	170 (93.4)	12 (6.6)	0 (0)
12. Benefit of exercise in optimiizing diabetes control	177 (97.3)	5 (2.7)	0 (0)
13. More care is needed when cutting their toenails	145 (79.7)	29 (15.9)	8 (4.4)
14. Medication for diabetes can have better outcome than diet or exercise	113 (62.1)	67 (36.8)	2 (1.1)
15. High risk of oral disease with diabetes (e.g., dental caries)	117 (64.3)	30 (16.5)	35 (19.2)
16. The harm of smoking for the gums is more in diabetes in comparision with general population	166 (91.2)	11 (6)	5 (2.7)
17. Knowing when and how to take medications?	180 (98.9)	2 (1.1)	0(0)
18. Diabetes likely to occur in obese individuals Obesity	124 (68.1)	26 (14.3)	32 (17.6)
19. Other risk factors include low physical activity	143 (78.6)	32 (17.6)	7 (3.6)
20. In some individuals, genetic factors can be involoved in diabetes mellitus	130 (71.4)	46 (25.3)	6 (3.3)

DN: don’t know.

**Table 3:** Diabetes-related attitude questions at baseline (n = 182).

Attitude Items	Agree	Neutral	Disagree
1. Fear of my diabetes.	44 (24.2)	8 (4.4)	130 (71.4)
2. Hard to believe that having diabetes.	46 (25.3)	3 (1.6)	133 (73.1)
3. Feeling unhappy and depressed because of diabetes.	43 (23.6)	24 (13.2)	115 (63.2)
4. Feeling satisfied with life.	150 (82.4)	27 (14.8)	5 (2.7)
5. Difficuly in coping with diabetes.	46 (25.3)	37 (20.3)	99 (54.4)
6. I am getting on with my life despite diabetes.	89 (48.9)	48 (26.4)	45 (24.7)
7. I think it is of signifcant to achieve the following:			
a. Good control of blood sugar.	179 (98.4)	1 (0.5)	2 (1.1)
b. Weight control.	174 (95.6)	7 (3.8)	1 (0.5)
c. Care for my diabetes (diet, medicine, exercise, etc.).	176 (96.7)	4 (2.2)	2 (1.1)
d. Awareness of my emotions and feelings (fear, worry, anger) about my diabetes.	164 (90.1)	16 (8.8)	2 (1.1)
8. I think I’m able to do the following:			
a. Good control of blood sugar.	118 (64.8)	16 (8.8)	48 (26.4)
b. Weight control.	105 (57.5)	20 (11)	57 (31.3)
c. Care for my diabetes (diet, medicine, exercise, etc.).	127 (69.8)	11 (6)	44 (24.2)
d. Cope well with my emotions and feelings (fear, worry, anger) about my diabetes.	95 (52.2)	28 (15.4)	59 (32.4)

DN: don’t know.

**Table 4:** Comparison of knowledge, attitude scores, and HbA1c levels at baseline and at 12 months (n = 182).

Variable		Baseline	12 Months	*p* Value
Knowledge scores	Mean (±SD)	14.8 (±2.5)	16.2 (±1.9)	<0.001
Knowledge	Low level	26 (14.3)	19 (10.6)	<0.001
	Moderate level	129 (70.9)	109 (60.9)	
	High level	27 (14.8)	51 (28.5)	
Mean change from baseline to 12 months	Mean (±SD)	1.4 (±0.1)		<0.001
Attitude scores	Mean (±SD)	21.7 (±3.3)	23.4 (±2.9)	0.015
Attitude	Negative attitude	34 (18.7)	30 (16.8)	0.032
	Fair attitude	115 (63.2)	130 (72.6)	
	Positive attitude	33 (18.1)	19 (10.6)	
Mean change from baseline to 12 months	Mean (±SD)	1.7 (±0.2)		<0.001
HbA1c (%)	Mean (±SD)	8.7 (±2.2)	6.8 (±0.8)	<0.001

## Data Availability

The data presented in this study are available on request from the corresponding author to keep the privacy of the participants.
